# Microbial fuel cell scale-up options: Performance evaluation of membrane (*c*-MFC) and membrane-less (*s*-MFC) systems under different feeding regimes

**DOI:** 10.1016/j.jpowsour.2021.230875

**Published:** 2022-02-01

**Authors:** Xavier Alexis Walter, Elena Madrid, Iwona Gajda, John Greenman, Ioannis Ieropoulos

**Affiliations:** a Bristol BioEnergy Centre, Bristol Robotics Laboratory, T-Block, UWE, Coldharbour Lane, Bristol, BS16 1QY, UK; b Biological, Biomedical and Analytical Sciences, UWE, Coldharbour Lane, Bristol, BS16 1QY, UK

**Keywords:** Microbial fuel cells, Scalability, Potentiostatic conditions, Artificial urine media, Energy conversion

## Abstract

In recent years, bioelectrochemical systems have advanced towards upscaling applications and tested during field trials, primarily for wastewater treatment. Amongst reported trials, two designs of urine-fed microbial fuel cells (MFCs) were tested successfully on a pilot scale as autonomous sanitation systems for decentralised area. These designs, known as ceramic MFCs (***c***-MFCs) and self-stratifying MFCs (***s***-MFC), have never been calibrated under similar conditions. Here, the most advanced versions of both designs were assembled and tested under similar feeding conditions. The performance and efficiency were evaluated under different hydraulic retention times (HRT), through chemical oxygen demand measures and polarisation experiments. Results show that ***c***-MFCs displayed constant performance independently from the HRT (32.2 ± 3.9 W m^−3^) whilst displaying high energy conversion efficiency at longer HRT (NER_*COD*_ = 2.092 ± 0.119 KWh.Kg_*COD*_^−1^, at 24h HRT). The ***s***-MFC showed a correlation between performance and HRT. The highest performance was reached under short HRT (69.7 ± 0.4 W m^−3^ at 3h HRT), but the energy conversion efficiency was constant independently from the HRT (0.338 ± 0.029 KWh.Kg_*COD*_^−1^). The ***c***-MFCs and ***s***-MFCs similarly showed the highest volumetric efficiency under long HRT (65h) with NER_*V*_ of 0.747 ± 0.010 KWh.m^−3^ and 0.825 ± 0.086 KWh.m^−3^, respectively. Overall, ***c***-MFCs seems more appropriate for longer HRT and ***s***-MFCs for shorter HRT.

## Highlights

• Most advanced ceramic and self-stratifying microbial fuel cells designs were tested.
• Ceramic microbial fuel cells have high energy conversion efficiency.
• Ceramic microbial fuel cells have constant power performance.
• Self-stratifying microbial fuel cells have constant energy conversion efficiency.
• Self-stratifying microbial fuel cells have higher power performance at short HRT.

## Graphical abstract

**Figure:**
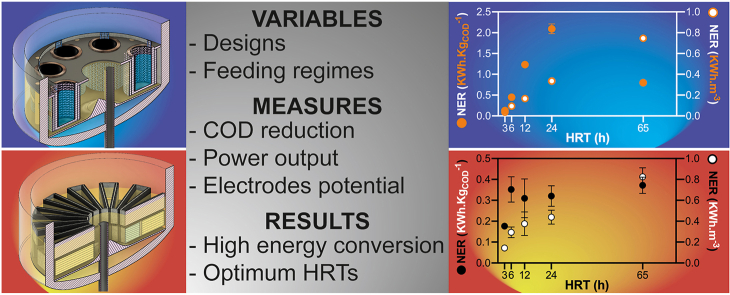

## 1 Introduction

In the context of reducing the impacts of human activities on the environment, the development of the microbial fuel cell technology, discovered in 1911 by M. C. Potter [1], has been gathering increasing interest since the beginning of the millennium [2,3]. This biotechnology exploits the capacity of certain anaerobic microorganisms to use conductive materials as the end terminal electron acceptor of their heterotrophic respiration, a capacity that has been recently shown to be widespread amongst the Bacteria and the Archaea domains [4,5]. When coupling such an electroactive, anaerobic, and heterotrophic respiration to the reduction of an oxidant, an electrical current is generated. Typically, a microbial fuel cell (MFC) reactor comprises an anodic compartment separated from a cathodic compartment by an ion exchange membrane or the body of electrolyte. In the anodic compartment, electroactive microorganisms mineralise organic substrates, with the anode electrode acting as the conduit for electrons released during their heterotrophic respiration. The electrons flow to the cathode, through an external circuit, where, in combination with cations e.g. protons that have diffused through the ion exchange membrane or electrolyte separating the two half-cells, they will be transferred to an oxidant, often oxygen, that gets reduced, thus completing the reactions/circuit and generating an electrical current.

The MFC technology is an energy transducer that converts the chemical energy contained in reduced organic matter into electrical energy. This principle opens numerous application avenues with the treatment of wastewater being the most investigated [6]. As the generated current is proportional to the biological activity, research investigates the use of MFCs as biosensors [7,8]. In addition, because of their electrochemical nature, MFCs are investigated as desalination apparatus [9,10], nutrient recovery systems [11], bioelectrolytic reactors [12,13], or selective depollution devices [14,15]. Overall, the wastewater treatment avenue has been considered to be the most promising in terms of applications [6]. Because human urine is responsible for 10% of the total chemical oxygen demand (COD), 75% of total nitrogen and 50% of phosphorous present in municipal wastewater [16,17], source separated sanitation and urine treatment has gained significant traction [18]. Logically, research has focused on developing systems focusing on the treatment of neat urine, and the MFC technology was shown to be a promising solution [11,19,20]. Research in the recent decade has made significant progress in the development of various urine treatment technologies. The industrialised process has not yet been established, but urine conversion technologies are at the point where commercial optimisation and market readiness is the next development step, taking into account that urine treatment should be as close as possible to the source to avoid transport [21] and further complicating the treatment process downstream. This gives a unique opportunity to harvest energy with the simultaneous waste treatment and recycling.

Recent field trials have demonstrated that autonomous sanitation systems could be deployed to simultaneously treat urine and generate energy to power lights [[22], [23], [24]]. Two different MFC designs have been employed for these trials, “two-chamber” ceramic based MFCs (***c***-MFC; [22,25]) and “single-chamber” stratifying MFCs (***s***-MFC; [23,26]). Both designs follow the same strategy of maximising the surface area of electroactive interfaces per volume of reactor through a miniaturisation approach [[27], [28], [29]]. However, each design has its specificity. The main difference however, is the presence or absence of the physical separator between the anodic and cathodic environments (half-cells) and the impact it has on treatment and operational power performance.

**Fig. 1:**
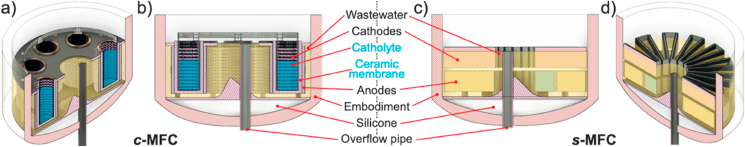
Isometric (**a**, **d**) and cross section (**b**, **c**) views of the ***c***-MFC (**a**, **b**) and ***s***-MFC modules (**c**, **d**).

The ***c***-MFCs have inexpensive ceramic membranes shown to be as effective as ion exchange membranes [30,31]. The most advanced ***c***-MFC modules comprise a multiplicity of small ceramic cylinders, sealed at the bottom with an external anode and an internal cathode (Fig. 1a and b; [32]). Having internal cathodes enables these setups to accumulate in the previously empty chamber an alkaline catholyte that has proven bactericidal properties [33,34] and other beneficial characteristics, currently being investigated. Hence, ***c***-MFCs can treat waste, generate energy, and produce a potential added-value product. Conversely, ***s***-MFCs type of design are characterised by the absence of a membrane, thus, anodes and cathodes sharing the same electrolyte. The ***s***-MFC design exploits the self-stratification phenomenon of water columns that results from the biological activity [29]. This design has been shown to be simple (Fig. 1c and d), inexpensive, scalable without performance losses, and amongst the most power-dense designs.

The present study continues investigating the MFC technology for urine treatment adding the latest development in the design of ***c***-MFCs and ***s***-MFCs. The use of optimised ceramic cylinders, electrode improvements (e.g. modification with activated carbon (AC) and better collectors) and new potentiostatic conditions were incorporated into the bioelectrochemical reactors of the present study. The context of the present study was to evaluate the efficiency and the performance of both designs under the same range of feeding regimes. This is to identify which design could be the most appropriate depending on the implementation conditions and application needs. Also, information gained will enable construction of multi-modular stacks specifically to the number of users by tailoring system characteristics to hydraulic retention time (HRT). Although the aim of the present study was to characterise the behaviour of two different systems under similar conditions, efforts were made for these two dissimilar types of MFC to have roughly similar parameters and operating conditions. The experiment comprised triplicates of each design, three ceramic MFCs (***c***-MFC) and three stratifying MFCs (***s***-MFC). The systems were fed with artificial urine medium (AUM) due to the unavailability of fresh urine because the ongoing SARS-CoV-2 pandemic and its implications during 2020–2021.

## 2 Experimental

### 2.1 Microbial fuel cell construction

#### 2.1.1 Ceramic microbial fuel cell

The *c*-MFC module comprised 8 individual units enclosed in the same cylindrical vessel (140 mm Plain PVC End Cap; Fig. 1a and b). Each individual MFC was built around a structural ceramic tube (50 mm height; 21 mm ID; 28 mm OD; Laufen) acting as the membrane. All 8 units were electrically connected in parallel. The cathodes were inserted inside each of the ceramic tubes. The cathode assembly comprised of a carbon veil (20 g m^−2^) coated with a 2 mm thick AC/PTFE (activated carbon (AC); polytetrafluoroethylene (PTFE)) mixture (80 wt% AC; 20 wt% PTFE) [35]. The cathode AC/PTFE loading was of 115 ± 3 mg cm^−2^. The cathode was not entirely in contact with the ceramic membrane due to an internal overlap and part of the cathode extending out of the ceramic tube. Therefore, it is considered here that the cathode surface area is equivalent to the internal surface area of the ceramic membrane, which corresponds to roughly 31 cm^2^ (21 mm ID; 47 mm height). Stainless steel mesh was introduced in the cathodic compartment and crimped onto the cathode to press the cathode against the ceramic membrane and to act as the current collector. The anode comprised a carbon veil (10 g m^−2^; 30 cm × 42 cm; 1260 cm^2^) folded down to 45 mm height, stapled (316 stainless-steel staples) onto a 316 stainless-steel mesh that also served as the current collector and wrapped on the external face of the ceramic tube. The carbon veil was covered by a mix of AC-PTFE (95 wt% AC; 5 wt% PTFE) to act as a catalyst [36]. The final AC-PTFE loading was of 1.25 ± 0.1 mg cm^−2^ (1575 ± 125 mg per anode). Since a module consisted of 8 ***c***-MFCs, the total anode surface area of the anode was 10,080 cm^2^ and 248 cm^2^, for the cathode, giving a an anode to cathode surface area ratio of 41:1 (Table 1). Due to the shape of the PVC embodiment, the bottom was filled with silicone to facilitate a flat bottom on which a 3D printed MFC support was placed. This support had a decentred flow diverter to maximise the flow distribution to all 8 MFCs within the module (Fig. 1a and b). Once the 3 replicate modules were assembled, the displacement volume, measured by weight, was equal to 435 ± 3 ml.

**Table 1:** Summary of the designs’ specifications.

Module type	Module volume	Electrolyte volume	Anode per module		Cathode per module		Number of MFC	MFC units	
			Surface area	AC/PTFE Loading	Surface area	AC/PTFE Loading		Anode	Cathode
***c***-MFC	2211 ml	435 ± 3 ml	10,080 cm^2^	1.25 ± 0.1 mg cm^−2^	248 cm^2^	115 ± 3 mg cm^−2^	8	30 × 42 cm	66*47 mm
***s***-MFC		525 ± 4 ml	10,080 cm^2^	1.19 ± 0.2 mg cm^−2^	263 cm^2^	186 ± 7 mg cm^−2^	28	30 × 12 cm	20*47 mm

#### 2.1.2 Stratified microbial fuel cell

The ***s***-MFC module comprised 28 anode-cathode pairs enclosed in the same cylindrical vessel (Fig. 1c and d). The cathodes and anodes were assembled on a 316 stainless-steel mesh concertina, with the cathodes positioned 5 mm above the anodes. The parallel electrical connection of all electrode pairs was integral to the built. The cathode assembly comprised a 2 mm thick AC/PTFE (activated carbon (AC); polytetrafluoroethylene (PTFE)) mixture (80 wt% AC; 20 wt% PTFE) pressed on a 316 stainless-steel mesh [37] (2 cm height by 155 cm length) that was folded as a concentric concertina (Fig. 1d). This mesh served as both a structural feature and a current collector. Each of the 28 cathodes (c.a. the part of the mesh covered with the AC/PTFE mix) was 20 mm in height and 47 mm in length for a surface area of 9.4 cm^2^. This 20 mm height was chosen because it was identified as the shallowest height prior to oxygen diffusion affecting the anodic layer [38]. Each cathode had an AC/PTFE loading of 186 ± 7 mg cm^−2^. The anode concertina comprised 28 carbon veils (10 g m^−2^; 30 cm × 12 cm; 360 cm^2^) folded down to 20 mm height and 47±2 mm length; stapled (316 stainless-steel staples) onto a 316 stainless-steel mesh (1.6 cm height by 155 cm length) that served as the current collector and was folded in concertina (Fig. 1c and d). The carbon veil was covered by a mix of AC-PTFE (95 wt% AC; 5 wt% PTFE) to act as a catalyst [36]. The final AC-PTFE loading was 1.19 ± 0.2 mg cm^−2^. Since a module comprised 28 anode/cathode pairs, a module had a total anode surface area of 10,080 cm^2^ and a total cathode surface area of 263 cm^2^, for an anode to cathode surface area ratio of 38 (Table 1). As for the ***s***-MFCs, the bottom of the PVC container was filled with silicone to manage a flat bottom on which was placed a 3D printed MFC support. The support had a decentred flow-diverter to maximise the flow distribution within the module (Fig. 1c and d). Once the 3 replicate modules were assembled, the displacement volume was measured by weight, and was equal to 525 ± 4 ml.

### 2.2 Operating conditions

All bioreactors were inoculated with a mixture made from 50% activated sludge and 50% (v/v) artificial urine media (AUM). The AUM media comprised: 5 g l^−1^ peptone, 2.5 g l^−1^ yeast extract, 5 g l^−1^ urea (85 mM), 5.2 g l^−1^ NaCl (90 mM), 3.2 g l^−1^ NaSO_4_.10H_2_O (10 mM), 0.95 g l^−1^ KH_2_PO_4_ (7 mM), and 1.2 g l^−1^ K_2_HPO_4_ (7 mM). After 2 weeks, the MFCs were fed with 100% AUM. The MFCs were fed under different hydraulic retention times (HRT) and the performance was recorded over time. The HRT applied to each MFC were 3h, 6h, 12h, 24h, 48h, and 65h. Since both types of MFC were fed in a continuous flow using the same peristaltic pump, the HRT differed slightly between the two designs. The correspondence to the exact HRT for each design can be found in Table 2. Each individual MFC module was fed by an independent stock solution of AUM.

**Table 2:** Specific retention time for each MFC design.

Mentioned HRT (h)	Electrolyte volume (ml)		Specific HRT (h)	
	*c*-MFC	*s*-MFC	*c*-MFC	*s*-MFC
3	435 ± 3	525 ± 4	2.7	3.3
6			5.5	6.6
12			10.9	13.2
24			21.9	26.4
48			43.8	52.9
65			56.8	68.5

### 2.3 Data capture and systems characterisation

From inoculation and throughout the duration of the experiment the MFCs were connected to purpose-built circuitry that maintained each cascade under potentiostatic conditions at 400 mV. More detail on the circuitry is reported in a previous study [38]. This setup allowed convertion of the measured current into voltage, which was recorded by an Agilent Data Acquisition System (Agilent LXI 34972A; Farnell, UK). Measurements were recorded every 5 min. The current *I* in Amperes (A) was calculated using conversion formula, *I =(V*_*m*_ *- 1.2)/19.8* [38], where *V* is the measured voltage in Volts (*V*_*m*_). The power output *P* in Watts (W) was calculated as *P = I×V*, where *V* is the constant voltage (400 mV) in Volts (V) and *I* the calculated current.

An initial cathode polarisation experiment was run prior to inoculation of the MFCs, 12h after being filled with AUM to avoid the presence of oxygen adsorbed on the activated carbon surface. The polarisation experiments were performed using linear sweep voltammetry (LSV) under a slow scan rate (0.25 mV s^−1^) to avoid overestimation of the performance. The potentiostat (Biologic SP50, France) was used in a three-electrode configuration. The anodes were used as counter electrodes, the cathodes as the working electrodes and an Ag/AgCl (3 M KCl) as a reference electrode. The reference electrode was placed next to the cathode to reduce the ohmic resistance given by the AUM [39,40].

Each MFC was characterised before shifting conditions from one HRT to the other. The characterisation consisted in running polarisation experiments and measuring the chemical oxygen demand (COD). The polarisation run of each MFC was performed running a linear sweep voltammetry (LSV) under a two-electrodes configuration. The reference electrode and counter leads were connected together to the cathode electrode. Meanwhile the working lead was connected to the anode electrode.

The potentials were also measured against the cathode electrode using a PicoTech data logger (ADC-24, Pico Technology Ltd) using an Ag/AgCl (3 M KCl) reference electrode. In *s*-MFC, the Ag/AgCl reference electrode was positioned within the liquid layer separating the cathode and anode concertina for it to be equidistant of both electrodes. In the *c*-MFC, the Ag/AgCl reference electrode was placed in the anodic chamber. This operation was done to obtain the polarisation curves of both anodes and cathodes separately. Also in this case, the scan rate was 0.25 mV s^−1^ and ranged from OCV to 50 mV.

The COD analyses were performed through the potassium dichromate oxidation method (COD HR test vials, Camlab, UK) with 0.2 mL of filtered (0.45 μm, Syringe Filter Millipore) inlet and outlet samples. The duplicate samples of the inlet were taken from the inlet tubing that was feeding each MFC. Output samples were taken from the outlet of each MFC. Each duplicate and triplicate data were averaged (i.e. ***c***-MFC and *s*-MFC). The results presented here show the average of the two distinct ***c***-MFC and ***s***-MFC designs.

## 3 Results & discussion

### 3.1 Cathode polarisation under clean conditions

**Fig. 2:**
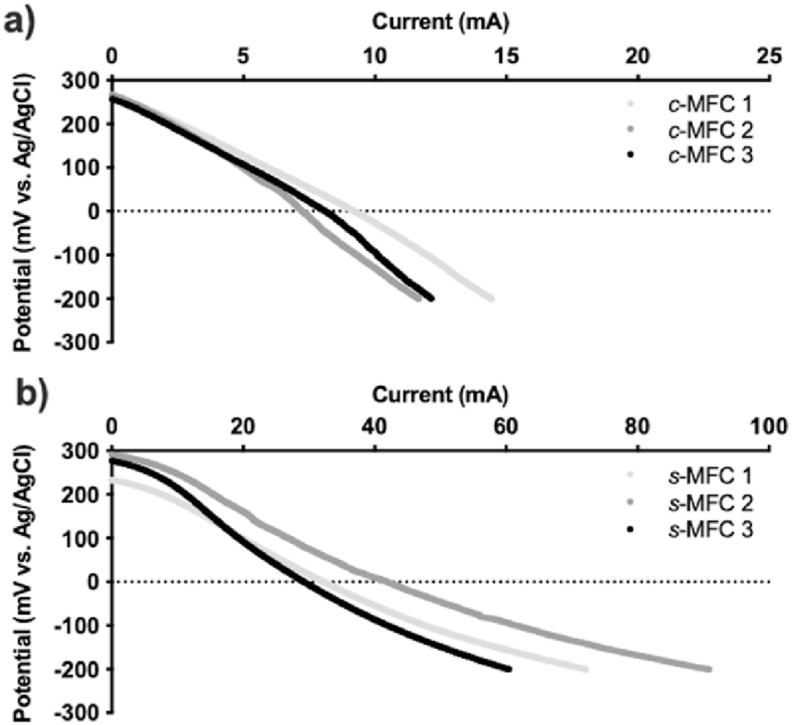
Initial cathodes polarisation of each ***c***-MFC module (**a**) and ***s***-MFC module (**b**).

The experimental setup was first characterised by an initial polarisation experiment of the uninoculated modules filled with fresh AUM (pH = 8.4; EC = 14.5 mS cm^−1^; Fig. 2). The LSV's were performed under a three-electrode configuration. However, due to the nature of the ***c***-MFC modules, made of 8 isolated cathodic compartments, the reference electrode was placed in the anodic chamber. This implies that the resistance of the ceramic membrane limits the measured performance of the cathodes. Nevertheless, the results give an overview of the impact of presence/absence of ceramic membrane and a direct comparison point to evaluate the evolution of the systems.

The cathodes of both designs had similar open circuit voltage levels with 263.6 ± 5.5 mV vs Ag/AgCl and 284.2 ± 10.0 mV vs Ag/AgCl for the ***c***-MFC and the ***s***-MFC, respectively. For current generation the cathodes of the ***c***-MFCs produced 6.1 ± 1.0 mA @ 0 mV vs Ag/AgCl, whilst the cathodes from the ***s***-MFCs displayed a greater variation between each replicate producing 25.8 ± 6.6 mA @ 0 mV vs Ag/AgCl. At a potential of 0 mV, this corresponds respectively to roughly 16% and 26% variation between replicates. Despite a greater variation, the cathode polarisation results of the ***s***-MFC (98 ± 25 μA cm^−2^ @ 0 mV vs Ag/AgCl) are similar to the ones reported in previous studies using similar electrode size (99 ± 7 μA cm^−2^ @ 0 mV vs Ag/AgCl) [37].

### 3.2 Evolution of the power output under different feeding regimes

**Fig. 3:**
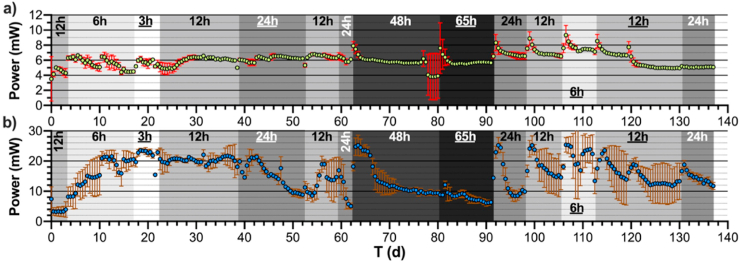
Evolution of the power output illustrating the impact the HRT has on the MFC design: (a) ***c***-MFCs and (**b**) ***s***-MFCs. The hours stand for the HRT of the modules. The underlined hours indicates when the polarisation experiments were undertaken.

Once inoculated, the MFC modules were fed with a mixture of activated sludge and AUM during 2 weeks with a HRT of 12h (data not shown). After this inoculation phase, the modules were fed solely with AUM at 12h HRT for 3 days (Day-0 in Fig. 3). At the end of this period, both module-designs were producing 4.3 ± 0.5 mW. The HRT was then shifted to 6h HRT. The following days ***c***-MFCs reach a dynamic steady state at around 6.4 ± 0.3 mW at Day-5 (Fig. 3a). Conversely, the ***s***-MFCs reached steady state on Day-11 at 21.5 ± 1.3 mW. These results suggest that at Day-11 both designs reached maturity (Fig. 3) since, the impact of the feeding regime on the behaviour of both designs differed greatly depending on the design. The feeding regimes were shifted to 48h HRT and then to 65h HRT during the winter 2020/21 UK-lockdown period resulting from the SARS-CoV-2 pandemic.

Along the 135 days of the experimental run, the ***c***-MFC modules displayed a stable power output independently from the feeding regime (Fig. 3a). Not only were the outputs stable throughout the experiment from day-to-day (6.3 ± 0.7 mW per ***c***-MFC) but also between the replicates with an average variation of ±0.3 mW. Although homogeneous, a maximum power output was reached during Day-105 to Day-112 when the ***c***-MFC modules were well matured, and the HRT set at 6h (7.4 ± 0.4 mW per *c*-MFC). This result suggests that full maturity of the ***c***-MFC was reached on Day-91. Interestingly, even under a feeding regime resulting in 48h and 65h HRT, the ***c***-MFC modules produced similar power levels that were roughly 6% lower (5.9 ± 0.9 mW). Once the feeding regimes were shifted to shorter HRT, the power output of the ***c***-MFC modules rapidly recovered (6.4 ± 1.2 mW per ***c***-MFC).

Over the course of the experimental run, the power output of the ***s***-MFC modules had displayed great variations, from 22.7 ± 0.9 mW (3h HRT; Day-18 to Day-22; Fig. 3b) down to 7.0 ± 0.6 mW (65h HRT; Day-87 to Day-91). These results suggest that there is a clear correlation between the feeding regime and the power output of *s*-MFC. Although correlated, the changes in power output were progressive from one steady state to another, especially when under HRT conditions of 12h or more. For example: it took 8 days for the ***s***-MFC to reach steady state under a 65h feeding regime (Day-82 to Day-89), 9 days under 48h HRT (Day-66 to Day-74), 6 days under 24h HRT (Day-44 t Day-49) and 4 days under 12h HRT (Day-54 to Day-57; Day-121 to Day-124). The scale of these changes suggests that the structure of the microbial communities changed as well, with 12h HRT being the tipping-point condition between two different community structures. Another clear feature shown by the results is the variability between replicates. The outputs from the ***s***-MFC could vary from ±0.1 mW (Day-28) to ±8.9 mW (Day-118), as indicated by error bars. This could be due to dynamic responses of the anodic and cathodic biofilm to flow rate.

### 3.3 Polarisation experiments on matured MFC depending on feeding regimes

Prior to changing feeding rates and subsequent HRTs, a polarisation experiment was carried out on each MFC module to characterise the state of each system after it had been running under each experimental condition: 3h HRT (Day-23; Fig. 3), 6h HRT (Day-113; Figs. 3), 12h HRT (Day-130; Figs. 3), 24h HRT (Day-52; Figs. 3) and 65h HRT (Day-92; Fig. 3). The objective was to measure the impact that the feeding regime had on the MFCs electrical performance. Before the polarisation analysis, the modules were maintained in OCV for 45 min.

**Fig. 4:**
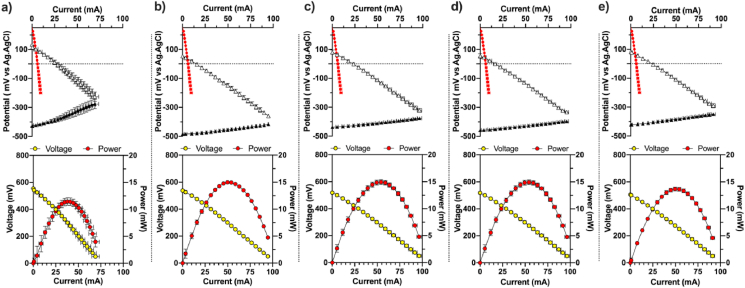
***c***-MFC electrodes polarisation under different running conditions: (**a**) 3h HRT; (**b**) 6h HRT, (**c**) 12h HRT, (**d**) 24h HRT, and (**e**) 65h HRT. The top graphics represent the average electrodes potentials. White triangles stand for the cathode potential, the black triangles stand for the anode potential and the red circles stand, as a reference, for the initial cathode potential (i.e. prior inoculation). The bottom graphics shows the average polarisation of the ***c***-MFC modules. (For interpretation of the references to colour in this figure legend, the reader is referred to the Web version of this article.)

Results from the electrodes polarisation of the ***c***-MFC modules show that compared to the initial cathode polarisation the open circuit potential (OCP) had decreased by 69%. The initial cathode OCP, prior to inoculation, was of 263.6 ± 5.5 mV vs Ag/AgCl, whereas the average cathodic OCP, under all running conditions, was of 81.1 ± 32.3 mV vs Ag/AgCl (Fig. 4). This result indicates that the cathodes were more reduced. On the other hand, the average current produced at 0 mV vs Ag/AgCl has increased by nearly 4-fold to 21.8 ± 5. mA (average of all conditions; Fig. 4a–e). Also, the slope of the cathode polarisation curves is higher than the anodes polarisation curves under all tested conditions (Fig. 4a–e). Such a result indicates that in the ***c***-MFC modules, the cathodes limit the overall performance. The results from the polarisation experiment of the 3h HRT condition (Fig. 4a) indicate that the maximum current produced was of 69.1 ± 4.2 mA. In comparison, the average maximum power produced by the ***c***-MFC modules under 6h, 12h, 24h and 65h HRT, was of 94.4 ± 2.2 mA. This difference suggests that the ***c***-MFC modules had not yet reached their full potential at the end of the 3h HRT condition (Day-23; Fig. 3). The 24h HRT condition results support this hypothesis with a maximum current of 95.4 ± 1.9 mA (Fig. 4d). Indeed, the 24h condition was investigated after the 3h one. Hence, it could be suggested that ***c***-MFCs had reached full maturity after the end of the 24h HRT condition, after Day-52 (Fig. 3). These results are in agreement with previous studies, under continuous flow, with maturity reached at 50 days [41]. Regarding the anode OCP of the ***c***-MFC modules, all conditions displayed similar values with an average of −445.5 ± 25.6 mV vs Ag/AgCl (Fig. 4a–e).

The modules were characterised through an overall polarisation curve in a classic two-electrode configuration. Although the open circuit voltage (OCV) was similar under all investigated conditions (527.2 ± 19.6 mV), the power curves show that the ***c***-MFCs were not fully mature when the 3h HRT condition was investigated (Fig. 4a). Compared to previous results (690 mV, [41]), the OCV values are lower. Combined with the results of the cathodes polarisation, it is suggested that either the oxygen diffusion towards the cathode or the diffusion of reduced anolyte through the membrane could be a factor limiting the performance of the cathodes in ***c***-MFC systems. Under the 3h HRT feeding regime, the maximum power was of 11.5 ± 0.7 mW whereas the other conditions reached an average maximum power of 14.6 ± 0.6 mW (Fig. 4b,c,d,e), which correspond to a 27% increase. This means that mature ***c***-MFC modules had the potential to deliver a power density 33.5 ± 1.4 W m^3^. This corresponds to an average power of 1.83 mW by a single ceramic MFC (i.e. 8 units per module). Overall, the results of the polarisation experiments performed on the ***c***-MFCs (Fig. 4) confirm the observations made on the evolution of the power during the 135 days of the experiment (Fig. 3); once matured the ***c***-MFC modules displayed the same characteristics independently from the feeding regime.

**Fig. 5:**
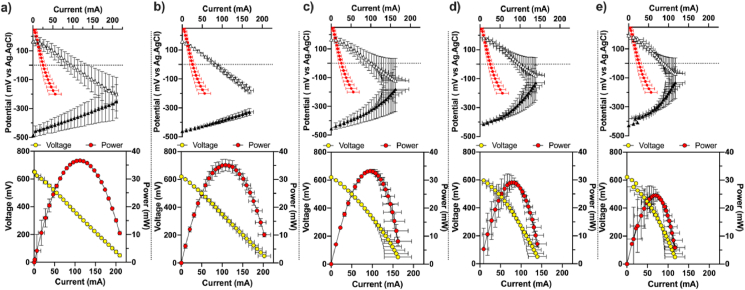
***s***-MFC electrodes polarisation under different running conditions: (**a**) 3h HRT; (**b**) 6h HRT, (**c**) 12h HRT, (**d**) 24h HRT, and (**e**) 65h HRT. The top graphics represent the average electrodes potentials. White triangles stand for the cathode potential, the black triangles stand for the anode potential and the red circles stand, as a reference, for the initial cathode potential (i.e. prior inoculation). The bottom graphics shows the average polarisation of the ***s***-MFC modules. (For interpretation of the references to colour in this figure legend, the reader is referred to the Web version of this article.)

Results from the electrode polarisation experiment show that the cathodes OCP of the ***s***-MFC modules (173.1 ± 14.0 mV vs Ag/AgCl; Fig. 5) had decreased by 39% compared to the initial cathode OCP (284.2 ± 10.0 mV vs Ag/AgCl; Fig. 2). Also, the cathode OCP stayed similar between all incubation conditions. Similarly, the anode OCPs of the ***s***-MFC modules stay comparable independently of the feeding regime (−449.1 ± 28.3 mV vs Ag/AgCl). In addition, the current produced when the cathodes were at 0 mV also stay comparable across all conditions (93.3 ± 7.5 mA). Compared to the initial polarisation (25.8 ± 6.6 mA @ 0 mV vs Ag/AgCl), these results correspond to a 3.5-fold increase. These results indicate that the open circuit states of the ***s***-MFC modules remained similar throughout the 135 days of the experiment. Although the slopes of the cathode and anode polarisation curves were comparable, the slopes of the cathode curves were slightly higher, thus, indicating that they were the factor limiting the systems. Interestingly, with increasing HRT conditions, the cathode slopes did not change (−2.14 ± 0.35) as much as the anode slopes (1.70 ± 0.82). Moreover, when considering the maximum current produced, the results indicate a decrease in electrocatalytic activity when the HRT increased from 3h, 6h, 12h, 24h and 65h (Fig. 5a–e); particularly, the maximum current produced was of 209.5 ± 3.5 mA, 162.5 ± 33.5 mA, 162.5 ± 33.5 mA, 139.4 ± 21.9 mA, 116.7 ± 24.5 mA, respectively. These results suggest that the electrocatalytic activity of the anodes was most affected by the changes in the feeding regime.

The overall polarisation experiments, in a classic two-electrode configuration, confirm the observations made from the electrode polarisation curves. As indicated by the constant OCP of the electrodes and except for the 65h HRT, the open circuit voltage of the ***s***-MFC modules (OCV) was constant throughout the experiment at around 615 ± 12 mV (Fig. 5). The 65h conditions displayed both a higher average OCV 688 ± 48 mV and a higher variation between replicates. In terms of the electrical performance, results indicate that the ***s***-MFC modules were affected by the feeding regime (Fig. 5). As shown by the polarisation curves, the maximum power produced is inversely correlated to the feeding regime with 3h HRT > 6h HRT > 12h HRT > 24 HRT > 65 HRT: 36.6 ± 0.2 mW > 35.2 ± 2.1 mW > 33.3 ± 0.4 mW > 30.0 ± 1.9 mW > 24.6 ± 2.2 mW, respectively (Fig. 5). When normalised by the displacement volume (525 ml), these values correspond to 70 W m^−3^, 67 W m^−3^, 63 W m^−3^, 57 W m^−3^ and 47 W m^−3^, respectively. Compared to the power densities reported in a previous study with ***s***-MFC having electrodes of the same height (27.8 ± 0.9 W m^−3^; 39h HRT [38]), these results are 50% higher at similar HRT (calculated ±55 mW m^−3^ at 39h HRT). This could be due to the present design having a 30% higher cathode surface area to volume ratio of 0.50 cm^2^ ml^−1^, whereas in the previous study the ratio was of 0.35 cm^2^ ml^−1^. Overall, the results of the polarisation experiment show a direct correlation between the electrocatalytic properties of ***s***-MFC modules and the feeding regime. The longer the retention time, the lower the electrochemical performance.

### 3.4 Performance under running conditions

Chemical oxygen demand (COD) measures on each MFC module were performed prior to changing feeding rates and subsequent HRTs. To characterise the treatment efficiency of each module under each feeding regime, filtered samples were taken on the inlets and outlets: 3h HRT (Day-23; Fig. 3), 6h HRT (Day-113; Figs. 3), 12h HRT (Day-130; Figs. 3), 24h HRT (Day-52; Figs. 3) and 65h HRT (Day-92; Fig. 3). The measures were then averaged for each design. As contaminations occurred in the feedstock supply tanks and tubing on the longer HRTs, different COD concentrations were measured at the inlet between feeding regimes (6.12 ± 1.04 g_COD_.l^−1^, n = 60). Hence, to have comparable data between each incubating condition, the COD results were converted as percentages, and the removal rates were reported as percentages of the inlet concentration.

**Fig. 6:**
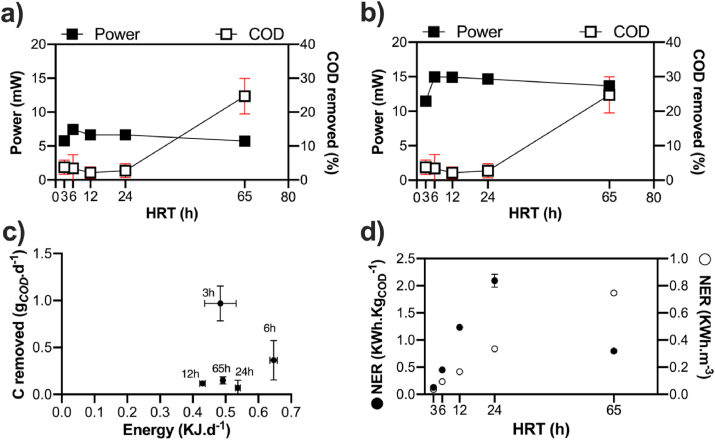
Relationship, for the ***c***-MFC, between the COD removal and the power output under running condition (**a**) or the maximum power output from the polarisation experiment (**b**). The power output under running conditions is the average of the last 48h prior the change of HRT. (**c**) Relation between the quantity of energy produce per day under “running conditions” (KJ.d^−1^) and the quantity of carbon removed per day (g_*COD*_.d^−1^). (**d**) Normalised energy recovery (NER) illustrating the energy conversion efficiency [42,43].

As for the electrocatalytic performance, the COD removal rates of the ***c***-MFC modules were similar for most feeding regimes. On average, the COD removal rates of the ***c***-MFC modules were 3.0 ± 0.7% for the 3h, 6h, 12h and 24h HRT conditions (Fig. 6a–b). Conversely to the other feeding regimes, the COD removal rates of the ***c***-MFC modules was of 24.7 ± 5.2% when having 65h HRT. Interestingly, this increase of the COD removal is not correlated to the power produced by ***c***-MFCs that displayed an output similar to previous HRT. Having a higher COD removal with increased HRT resulted in previous studies in a lower current output when compared to shorter HRT. This decorrelation could be due to the fact that the carbon loading was yet sufficient to sustain a similar microbial electroactivity. A result that supports this hypothesis is the carbon removal rate of the 65h HRT condition that is similar to other conditions (Fig. 6c; 0.150 ± 0.039 g_*COD*_.d^−1^). Regardless of the maximum power from the polarisation experiment (Fig. 6b; 14.0 ± 1.7 mW, 32.2 ± 3.9 W m^−3^) or the average power produced during the last 48h of each condition (Fig. 6a; 6.6 ± 0.7 mW; 15.2 ± 1.6 W m^−3^), the ***c***-MFCs produced roughly the same power independently from the feeding regime. Although treating real urine, previous studies on ceramic MFC reported lower power densities (21 W m^−3^; [36]) but higher COD removal rates at both 24h HRT (±40%; [36,41]) or 72h HRT (±65%; [36,41]). The source of discrepancy between this study and the previous ones has not been identified. A possible avenue to investigate could be the differences brought by the feedstock and its subsequent impact on the microbial communities. Another difference that could play a role is that the systems were placed under continuous feeding in the present study whereas previous work was performed under batch-fed conditions. Regarding the difference in power output levels between the long-term run (Fig. 6a) and the polarisation experiment (Fig. 6b), results indicate that the c-MFC modules were producing roughly 53% less power independently from the feeding regime.

To have a better understanding of the ***c***-MFCs performance, the power output and the COD removal measures were converted in energy produced per day and in quantity of COD-carbon removed per day. As shown in Fig. 6c, the best performance of the ***c***-MFCs modules was reached under the 3h HRT conditions (0.484 ± 0.048 kJ d^−1^, 0.967 ± 0.185 g_*COD*_.d^−1^). However, the performance of the 3h HRT implies that more volume will be needed to produce the energy and proportionally less carbon will be removed per unit of wastewater volume. In terms of efficiency, the normalised energy recovery (NER) [42,43] is accepted as a good parameter to compare dissimilar bioelectrochemical systems that aim at converting organic substrate from wastewater into electricity. NER normalises the energy extracted either by the amount of COD removed (NER_*COD*_: KWh.Kg_*COD*_^−1^) or by the volume of waste treated (NER_*V*_: KWh.m^−3^) using the equations:

(1) $$NER_{COD}\;\left[KWh.Kg_{COD}-1\right]=\;\frac{P\;\left[KW\right]\;\times \;t_{treatment}\;\left[h\right]}{V_{Treated\;during\;t}\left[m^{3}\right]\;\times \;\Delta COD\;\left[Kg_{COD}.m^{-3}\right]}$$

(2) $$NER_{V}\;\left[KWh.m-3\right]=\;\frac{P\;\left[KW\right]\;\times \;t_{treatment}\;\left[h\right]}{V_{Treated\;during\;t}\left[m^{3}\right]}$$

The efficiency of the energy conversion from carbon to electricity (Fig. 6d) indicates that ***c***-MFC modules reached an optimum at 24h HRT (NER_*COD*_ = 2.0924 ± 0.119 KWh.Kg_*COD*_^−1^). Moreover, the NER_*COD*_ shows that the efficiency continuously increases from 3h HRT to 24h HRT (Fig. 6d). This result reflects the constant power production and COD removal rate, independently from the feeding regime, of the ***c***-MFC. Normalising the energy recovery per unit of volume (NER_*V*_) shows a positive correlation with the HRT. The longest HRT condition has the highest value (0.747 ± 0.010 KWh.m^−3^; Fig. 6d). This result is in agreement with the nature of the MFC technology. The longer a given wastewater volume stays in the system, the more carbon gets removed, and the more energy is produced. This is valid as long as the wastewater contains microbiologically accessible carbon. Overall, these results suggest that the optimum balance between performance (Fig. 6c) and efficiency (Fig. 6d) is found at 24 h HRT (0.537 ± 0.008 kJ d^−1^; 0.070 ± 0.081 g_*COD*_.d^−1^ removed; NER_*COD*_ = 2.092 ± 0.119 KWh.Kg_*COD*_^−1^; NER_*V*_ = 0.335 ± 0.019 KWh.m^−3^). Compared to other studies, these results are similar to the maximum reported (NER_v_ = 1.35 KWh kg^−1^ COD; 0.38 KWh m^−3^; [44]). Moreover, the maximum NER_*V*_ reached by the ***c***-MFC modules under the 65h HRT condition (0.747 ± 0.010 KWh m^−3^) is much higher.

In terms of the potential for such *c*-MFC to power a practical application, two estimated stacks can be envisaged. The first potential scenario would be to employ *c*-MFC modules to fully charge a modern day smart-phone with an average battery capacity of 3500 mAh at 3.8 V. For this scenario, assuming 6h HRT, a single module would produce 0.646 kJ d^−1^ (Fig. 6c; 0.179 Wh.d^−1^). This corresponds to 47.1 mAh.d^−1^, which implies that 74 modules would be needed to fully charge a 3500 mAh battery once a day. The second potential scenario would be to power the lights of an autonomous 9-person urinal, such as the one previously described [23]. In this scenario, six 2.65 V LED strips would require 960 mA (2.544 W) 12h per day during night time (30.5 Wh.d^−1^). Assuming the 6h HRT performance, the stack would need to comprise 170 *c*-MFC modules.

**Fig. 7:**
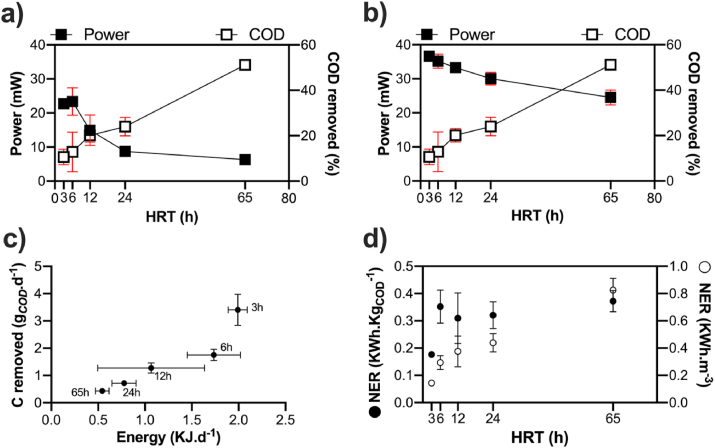
Relationship, for the ***s***-MFC, between the COD removal and the power output under running condition (**a**) or the maximum power output from the polarisation experiment (**b**). The power output under running conditions is the average of the last 48h prior the change of HRT. (**c**) Relation between the quantity of energy produce per day under “running conditions” (KJ.d^−1^) and the quantity of carbon removed per day (g_*COD*_.d^−1^). (**d**) Normalised energy recovery (NER) illustrating the energy conversion efficiency [42,43].

Conversely to the ***c***-MFC modules and as indicated by the polarisation experiments, the ***s***-MFC modules displayed a clear correlation between the power produced and the feeding regimes. When looking at the power produced under running conditions (Fig. 7a), results show a decrease of power with the increase of HRT. Interestingly, if the power decrease follows a one-phase decay profile, the COD removal rate increases with increasing HRT (Fig. 7a and b) in a somewhat linear correlation. Another result is the similar power output of the 3h HRT and 6h HRT conditions (Fig. 7a). This result could either be due to the immaturity of the ***s***-MFC modules on Day-23 or be an artefact of analysis due to the variation between replicates. When comparing the power output results between the “running conditions” and the “polarisation” experiments, the differences decrease with increasing HRT: 62% (3h HRT), 66% (6h HRT), 45% (12h HRT), 29% (24h HRT) and 26% (65h HRT). In either case, results indicate that the power output and the COD removal rate are inversely correlated with treatment efficiency and a decrease of the power output occurs when the HRT increase (Fig. 7a and b). When focusing on running conditions (Fig. 7a), the relationship between the treatment efficiency and the energy output seems to be balanced at 12h HRT with an average of 15.0 ± 4.4 mW and 20% COD removal per ***s***-MFC module. In previous studies where urine was fuelled to the reactors, a 12h HRT equate to a 45% COD removal at comparable working concentration. This suggests that AUM feedstock is harder to electroactively mineralise than neat urine.

As shown in Fig. 7c, there is a positive correlation between the energy produced by the ***s***-MFC and the carbon removed, with a maximum performance at 3h HRT condition (1.991 ± 0.101 kJ d^−1^; 3.402 ± 0.571 g_*COD*_.d^−1^) and a minimum performance under 65h HRT condition (0.544 ± 0.072 kJ d^−1^; 0.430 ± 0.047 g_*COD*_.d^−1^). This correlation illustrates that the best performance is achieved with lower HRT condition, but more volume of waste will be needed to reach this performance. This is illustrated with the lowest energy conversion from carbon to electricity (NER_*COD*_ = 0.177 ± 0.08 KWh.Kg_*COD*_^−1^; Fig. 7d) displayed by the 3h HRT condition. Beside the 3h HRT, all other conditions displayed a similar efficiency of 0.338 ± 0.029 KWh.Kg_*COD*_^−1^ (Fig. 7d). This result indicates that the ***s***-MFCs conversion efficiency was stable and independent from the feeding regime once maturity was reached (after D-23; Fig. 7a). Normalising the efficiency recovery per unit of volume (NER_*V*_) show a positive correlation with the HRT. The longest HRT condition has the highest value (0.825 ± 0.086 KWh.m^−3^; Fig. 7d), whilst the shorter HRT has the lowest value (0.143 ± 0.006 KWh.m^−3^; Fig. 7d). Combined with the performance, these results suggest that the optimum balance between performance (Fig. 7c) and efficiency (Fig. 7d) is found at 12 h HRT (1.066 ± 0.571 kJ d^−1^; 1.277 ± 0.192 g_*COD*_.d^−1^ removed; NER_*COD*_ = 0.309 ± 0.093 KWh.Kg_*COD*_^−1^; NER_*V*_ = 0.376 ± 0.112 KWh.m^−3^). Compared to other studies, these results are above the average (NER_v_ < 0.1 KWh m^−3^; NER_COD_ < 0.1 KWh kg^−1^COD; [42]), but lower than the maximum (NER_v_ = 1.35 KWh kg^−1^ COD; [44]). Although not being an optimal feeding regime, the 65h HRT displayed a NER_*V*_ twice higher (0.825 ± 0.086 KWh m^−3^) than the maximum reported [44].

Regarding a practical application, two potential scenarios can be envisaged. The first potential scenario would employ a *s*-MFC stack to fully charge a modern day smart-phone. In this scenario, assuming 6h HRT, a single module produces 1.735 kJ d^−1^ (Fig. 7c; 0.482 Wh.d^−1^). This corresponds to 126.8 mAh.d^−1^ and implies that a 28-modules stack would fully charge a 3500 mAh battery once a day. Assuming the same performance at 6h HRT, the second application scenario would see a stack of 63 *s*-MFC modules powering a 2.544 W lighting during night-time (12h).

## 4 Conclusion

Many comparative studies in the MFC field set out to propose solutions for technology improvement in terms of power density or COD reduction capability. However, the variability of experimental conditions between studies renders the comparison difficult even if normalised values are reported. The present study focused on two different low-cost and scalable approaches towards technology implementation that have been successfully deployed in the field applications. Although developed in the same environment and implemented in similar pilot-scale systems (ca. autonomous sanitation systems for decentralised area), these two designs were always studied under different running conditions. As these designs are different, in terms of the presence/absence of the membrane and consequent functionalities, their comparison is a challenging task but one that might aid technology deployment and applicability. Therefore, evaluating the performance of each design under similar incubating conditions is valuable since it enables calibrating each design, thus, evaluating which design could be favoured for specific applications. Results have shown that both designs had improved performance and efficiency compared to previous iterations. The ***c***-MFCs took around 50 days to reach full maturity and displayed similar output levels between replicates. Since the power performance levels of ***c***-MFCs were constant (power density of 32.2 ± 3.9 W m^−3^) independently from the feeding regimes, their normalised energy conversion efficiency increased with longer HRT. The ***c***-MFCs seems to reach an optimum balance between performance and efficiency with 24h HRT. Under this feeding regime, ***c***-MFCs displayed an energy conversion efficiency of 2.092 ± 0.119 KWh.Kg_*COD*_^−1^ and volumetric extraction efficiency of NER_*V*_ = 0.335 ± 0.019 KWh.m^−3^. The ***s***-MFCs took around 25 days to reach full maturity and displayed great variation between replicates depending on the feeding regimes. The ***s***-MFCs performance levels were also correlated across the feeding regimes, with a maximum at 3h HRT (power density of 69.7 ± 0.4 W m^−3^) and a minimum at 65h HRT (power density of 46.9 ± 4.2 W m^−3^). Conversely to the ***c***-MFCs, the ***s***-MFCs displayed similar energy conversion efficiency independent of the feeding regimes (0.338 ± 0.029 KWh.Kg_*COD*_^−1^). The observed difference of maturation time and output stability between the two designs could be due to the presence or absence of a membrane. A hypothesis, worth investigating further, would be that the lower diffusion resulting from the presence of a membrane slows the establishment of a stable microbial consortium. On the other hand, this lower diffusion between compartments could be the reason for the observed stable electrical output due to a more resilient bioelectrochemical environment in dual compartment MFC.

## CRediT authorship contribution statement

**Xavier Alexis Walter:** Supervision, Conceptualisation, Methodology, Investigation, Data curation, Formal analysis, Analysis and Interpretation, Writing – original draft, Writing – review & editing. **Elena Madrid:** Investigation, Formal analysis, Analysis and Interpretation, Writing – review & editing. **Iwona Gajda:** Methodology, Formal analysis, Analysis and Interpretation, Writing – review & editing. **John Greenman:** Supervision, Writing – review & editing. **Ioannis Ieropoulos:** Principal Investigator, Funding acquisition, Supervision, Writing – review & editing.

## Declaration of competing interest

The authors declare that they have no known competing financial interests or personal relationships that could have appeared to influence the work reported in this paper.
